# Effect of Time to Surgery of Colorectal Liver Metastases on Survival

**DOI:** 10.1007/s12029-020-00372-5

**Published:** 2020-02-22

**Authors:** Emerson Y. Chen, Skye C. Mayo, Thomas Sutton, Matthew R. Kearney, Adel Kardosh, Gina M. Vaccaro, Kevin G. Billingsley, Charles D. Lopez

**Affiliations:** 1 Division of Hematology Oncology, Knight Cancer Institute, Oregon Health & Science University, 3181 SW Sam Jackson Park Road, OC14HO, Portland, OR 97239 USA; 2 Division of Surgical Oncology, Knight Cancer Institute, Oregon Health & Science University, Portland, OR 97239 USA

**Keywords:** Colorectal cancer, Colorectal liver metastases, Chemotherapy, Clinical risk score, Time to surgery

## Abstract

**Purpose:**

Resection of liver-only colorectal liver metastases (CRLM) with perioperative chemotherapy is potentially curative. Specific primary tumor and liver metastasis characteristics have been validated to estimate the risk of recurrence. We hypothesize that the time interval from diagnosis of CRLM to surgery, or time to surgery (TTS), is clinically prognostic.

**Methods:**

Patients from a prospectively maintained institutional database at a Comprehensive Cancer Center from May 2003 to January 2018 were reviewed. Clinicopathologic, perioperative treatment, and TTS data were collected. TTS was categorized into short (< 3 months), intermediate (3–6 months), and long (> 6 months) intervals.

**Results:**

Two hundred eighty-one patients were identified. While overall survival (OS) was similar across TTS, postoperative overall survival (postoperative OS) of long TTS was associated with worse survival, 44 months (95% CI, 34–52) compared to short TTS, 59 months (95% CI, 43–79), and intermediate TTS, 63 months (95% CI, 52–108), both *p* < 0.01. With regard to long-term OS, intermediate TTS had 5-year OS of 59% and 8-year OS of 43% compared to long TTS (5-year OS 53% and 8-year OS 18%) and short TTS (5-year OS 54% and 8-year OS 29%). Long TTS was negatively associated with postoperative OS on multivariate analysis (HR 1.6, *p* < 0.01) when adjusting for resection margin, CRLM size, age, and use of postoperative chemotherapy.

**Conclusion:**

Short and intermediate TTS had similar survival although patients with intermediate TTS may have better odds of long-term OS. While long TTS was associated with worse survival, likely due to higher disease burden, long-term survivors were still observed.

**Electronic supplementary material:**

The online version of this article (10.1007/s12029-020-00372-5) contains supplementary material, which is available to authorized users.

## Background

Colorectal cancer (CRC) is the third most common type of adult cancer in the USA, causing an estimated 51,020 deaths in the USA in 2019 [1]. At diagnosis, 20–25% of patients with CRC will already have metastatic disease, most commonly to the liver [2]. Likewise, liver metastases are the most common recurrence site after initial resection of colorectal primary, ranging from 8 to 18% of cases [3–5]. Without surgical intervention and modern systemic therapies, colorectal liver metastases (CRLM) have a dismal prognosis, with a historic 5-year survival of 5% or less [6, 7]. However, multi-agent chemotherapy now has been shown to extend survival beyond 2 years [8], though it is still considered non-curative. If patients with liver-limited CRLM undergo definitive liver resection and perioperative chemotherapy, more than 20% are disease-free after 5 years [9]. Although the European Organization for Research and Treatment of Cancer (EORTC) 40983 randomized phase 3 trial was unable to confirm an overall 5-year survival benefit of perioperative FOLFOX4 chemotherapy with definitive liver resection, nevertheless, such clinical practice has since been widely adopted as standard of care, owing to a clear recurrence-free survival advantage [9, 10]. However, the optimal timing of liver resection remains unknown, as no randomized-controlled trials have compared neoadjuvant versus adjuvant chemotherapy or different durations of preoperative chemotherapy.

A scoring system has been developed to predict recurrence after liver resection for CRLM to better select patients who will benefit from definitive surgery [11]. Some patients with CRLM, despite baseline scan showing liver-only metastases, may develop additional metastatic disease during their treatment course. Many predictive factors, such as primary tumor location, synchronous vs. metachronous liver metastases, CEA level, size of liver metastases, number of liver lesions, type of preoperative chemotherapy, and extra-hepatic involvement, have all been studied with varying prognostic certainty [12, 13]. The decision to proceed with liver resection remains a discussion among experienced surgical oncologists, radiologists, and medical oncologists rather than by empirical evidence [14]. The timing of liver resection within 3 months of diagnosis was previously established in those with immediately resectable CRLM [15]. However, to our knowledge, the timing of liver resection across all patients with CRLM of varying disease burden is unknown and has not yet been correlated with long-term survival beyond 5 years.

We hypothesize that time to surgery (TTS), defined by initial diagnosis of CRLM to date of first definitive liver resection, correlates with recurrence-free survival (RFS) and overall survival (OS). This knowledge might help clinicians estimate the potential benefit of definitive liver resection.

## Methods

### Study Design and Population

A retrospective study of patients who had definitive liver resection of colorectal liver metastases (CRLM) between January 2003 and December 2017 at Oregon Health & Science University (OHSU) was conducted through electronic medical chart review up to January 2018. Patients were initially identified from a prospectively maintained clinical database that recorded all liver operations with a cancer diagnosis at OHSU. All adults diagnosed with CRLM and having definitive liver resection at OHSU were included in this investigation. The study protocol was approved by local institutional review board and was conducted in accordance with the Declaration of Helsinki.

### Data Extraction

Demographics, primary tumor pathology, liver metastasis characteristics, treatment details, and oncologic outcomes were extracted from electronic medical charts. All potentially predictive variables were chosen on the basis of commonly reported factors (e.g., age, race), previously validated factors (e.g., AJCC cancer stage, size of liver metastasis, primary tumor location), relevant treatment detail (e.g., oxaliplatin-based chemotherapy, irinotecan-based chemotherapy), and hypothesized variable of interest, TTS. TTS was defined by the time period from the diagnosis of CRLM to the date of first definitive liver resection. TTS was also categorized into short (less than 3 months), intermediate (3 months to less than 6 months), and long (6 months or greater) with the intention of categorizing patients with diverse treatment history into “no or minimal preoperative therapy,” “perioperative therapy,” and “total neoadjuvant therapy.” No upper limit of TTS was set because longer duration of chemotherapy does not typically prohibit definitive liver resection. Data for this project were stored in Oregon Clinical & Translational Research Institute’s installation of REDCap [16], a secure electronic research data collection and management system.

### Endpoints

Patients were retrospectively followed until lost to follow-up, death, or data cutoff date January 31, 2018. For univariate analysis, patients were categorized as (1) no evidence of disease (NED) if they were disease-free and had at least 12 months of follow-up or (2) disease recurrence or death if such event occurred during their follow-up. Ninety-day postoperative mortality was also recorded. OS was defined from discovery of initial CRLM to death to minimize lead time bias inherent in postoperative endpoints. Postoperative overall survival (postoperative OS) was defined by time from date of first definitive liver resection to death to minimize immortal time bias inherent in OS because TTS is a time interval included from discovery of CRLM to death. Postoperative recurrence-free survival (RFS) was defined from the first definitive liver resection to recurrence. All three survival endpoints therefore were deemed complementary due to each of their inherent biases. Missing outcomes were minimized by also searching through published obituaries and databases open to the general public.

### Statistical Analysis

Kaplan-Meier curve stratified by TTS was generated for OS (discovery of CRLM to death), postoperative OS, and RFS, and comparison was made using the log rank test. TTS and all collected variables were compared between those who were NED vs. recurrence/death using Fisher exact test for categorical variables and Mann-Whitney test for continuous variables. Variables with significance *p* < 0.20 were then selected for multivariate analysis using Cox proportional hazards test as the primary analysis to predict postoperative OS and sensitivity analysis to predict OS and RFS. The Cox proportional hazards test reported hazard ratio with regard to OS, postoperative OS, and RFS endpoints and was conducted in stepwise fashion with *p* < 0.05 to remain in the model but required the variable of interest, TTS, to stay in the final model regardless of significance. All analysis was generated using SAS software version 9.4, and figures were graphically edited using Microsoft PowerPoint.

## Results

### Clinicopathologic Characteristics

Two hundred eighty-one (*n* = 281) patients were identified with a median follow-up of 39 months (range 0.8–175) (Table 1). Median age was 62 years old (range 20–88), and 120 (43%) were female. Synchronous metastases were diagnosed in 165 (59%) patients. With regard to liver metastasis distribution, 38 (14%) had unilateral left liver metastases, 125 (44%) had unilateral right liver metastases, and 118 (42%) had bilateral liver metastases. The majority of patients had 3 or fewer liver lesions. The median diameter of dominant liver lesion was 3.6 cm (range 0.2–21). With regard to diagnostic workup, all patients had contrast CT, but 55 (20%) also had a preoperative liver MRI prior to resection, and 217 (77%) had PET scan done prior to liver resection.

**Table 1:** Baseline and treatment characteristics of study population

*N* = 281	*N* (%) or median (range)
*Age* (years)	62 (20–88)
*Sex*	
Female	120 (43%)
Male	161 (57%)
*Initial AJCC stage*, *N* = 279	
Stage I and II	53 (19%)
Stage III	61 (22%)
Stage IV	165 (59%)
*Primary tumor*, *N* = 280	
Right-sided	91 (33%)
Left-sided	189 (67%)
*Liver metastases*	
Synchronous	165 (59%)
Metachronous	116 (41%)
Left liver only	38 (14%)
Right liver only	125 (44%)
Bilateral liver	118 (42%)
Number of lesions, *N* = 280	2 (1–10^**a**^)
Diameter of dominant lesion (cm)	3.6 (0.2–21)
*Baseline CEA* (ng/mL), *N* = 174	9.8 (0.5–1847)
*Tumor molecular testing*	
KRAS or NRAS mutation, *N* = 163	62 (38%)
BRAF mutation, *N* = 99	5 (5.1%)
MSI-high status, *N* = 85	8 (9.4%)
*Specific diagnostics*	
Preoperative MRI abdomen	55 (20%)
Preoperative PET-CT	217 (77%)
*Time to surgery (TTS)*	
Short (< 3 months)	64 (23%)
Intermediate (3–6 months)	159 (34%)
Long (> 6 months)	122 (43%)
*Preoperative chemotherapy*	214 (76%)
Oxaliplatin-based only	146 (52%)
Irinotecan-based only	22 (7.8%)
Both oxaliplatin and irinotecan	34 (12%)
Anti-VEGF biologic, *N* = 280	103 (37%)
Anti-EGFR biologic, *N* = 280	18 (6.4%)
Any response to chemotherapy, *N* = 214	171 (80%)
*Postoperative chemotherapy*, *N* = 274	175 (64%)
*Positive resection margin (R1/R2)*	32 (11%)
*90-day postoperative mortality*	5 (1.8%)
*Median follow-up for RFS and postoperative OS* (months)	29 (0.2–169)
*Median follow-up for OS* (months)	39 (0.8–175)

^a^10 includes ten or more lesions

AJCC, American Joint Commission on Cancer; CEA, carcinoembryonic antigen; MSI, microsatellite instability; MRI, magnetic resonance imaging; PET, positron emission tomography; VEGF, vascular endothelial growth factor; EGFR, epidermal growth factor receptor; RFS, recurrence-free survival; postoperative OS, postoperative overall survival; OS, overall survival

### Treatment Characteristics

Oxaliplatin-based chemotherapy was most frequently used in patients receiving preoperative chemotherapy (146 [52%], Table 1). Thirty-four (12%) required both oxaliplatin-based and irinotecan-based regimens. Sixty-seven (24%) patients did not receive any preoperative chemotherapy. At least partial response was noted in 171 of 214 (80%) patients who received any preoperative chemotherapy. One hundred seventy-five of 274 (62%) patients received postoperative chemotherapy. Positive resection margin, including R1 and R2 resection, was noted in 26 (9%) and 6 (2%), respectively, of 281 patients. Among the 32 patients with R1 or R2 resection, 26 had preoperative chemotherapy, and 6 did not. Five of those 6 patients did undergo postoperative chemotherapy. The 90-day postoperative mortality was noted in 5 (1.8%) of the study population. Median TTS was 5.3 months (range 0–78), and 64 (23%) had short TTS, 159 (34%) had intermediate TTS, and 122 (43%) had long TTS.

### Survival Outcomes by TTS

**Fig. 1:**
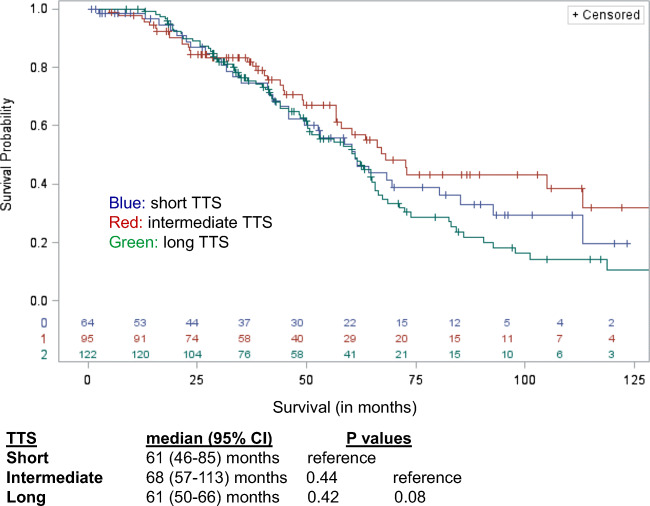
Intermediate TTS trend toward better OS with 68 months (95% CI, 57–113) compared to long TTS, 61 months (95% CI, 50–66). *p* = 0.08

When comparing OS from initial CRLM diagnosis to death across TTS, intermediate TTS had a trend toward better OS with 68 months (95% CI, 57–113) compared to long TTS, 61 months (95% CI, 50–66), *p* = 0.08 (Fig. 1). Otherwise, median OS was similar among all three TTS intervals. However, with regard to survival, intermediate TTS had 5-year OS of 59% and 8-year OS of 43% compared to long TTS (5-year OS 53% and 8-year OS 18%) and short TTS (5-year OS 54% and 8-year OS 29%).

**Fig. 2:**
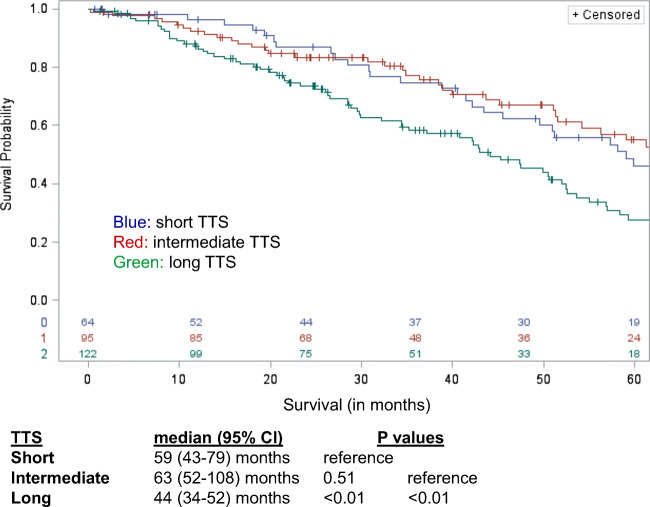
Long TTS associated with worse survival, 44 months (95% CI 34–52) compared to short TTS, 59 months (95% CI 43–79), and intermediate TTS, 63 months (95% CI 52–108). Both *p* < 0.01

When comparing postoperative OS from liver resection to death across TTS, long TTS was associated with worse survival, 44 months (95% CI 34–52) compared to short TTS, 59 months (95% CI 43–79), and intermediate TTS, 63 months (95% CI 52–108), both *p* < 0.01 (Fig. 2). With regard to postoperative long-term survival, 5-year postoperative OS in long TTS was 28% compared to short TTS (46%) and intermediate TTS (55%).

**Fig. 3:**
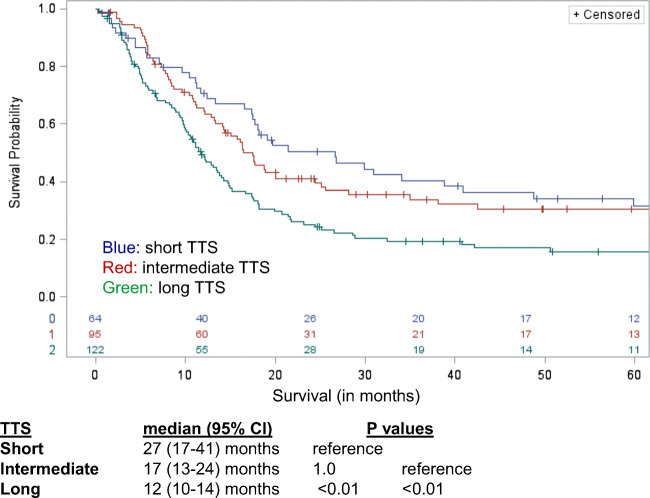
Long TTS associated with worse RFS, 12 months (95% CI, 10–14) compared to short TTS, 27 months (95% CI, 17–41), and intermediate TTS, 17 months (95% CI, 13–24). Both *p* < 0.01

Similarly looking at postoperative RFS, long TTS was associated with worse RFS, 12 months (95% CI, 10–14) compared to short TTS, 27 months (95% CI, 17–41), and intermediate TTS, 17 months (95% CI, 13–24), both *p* < 0.01 (Fig. 3).

### Predictors of Survival

On multivariate analysis, TTS ≥ 6 months was negatively associated with postoperative OS, (HR 1.6, *p* < 0.01), along with positive margins (HR 1.8, *p* < 0.01), diameter of dominant liver lesion (HR 1.1, *p* < 0.05), and age (HR 1.02, *p* < 0.05, Table 2). Treatment with postoperative chemotherapy was positively associated with postoperative OS (HR 0.63, *p* < 0.05). Sensitivity analysis, done to predict OS, confirmed positive resection margin (HR 1.7, *p* < 0.01), dominant liver lesion diameter (HR 1.1, *p* < 0.05), and treatment with postoperative chemotherapy (HR 0.63, p < 0.05) but not age nor TTS ≥ 6 months (HR 1.1, *p* = 0.55, Table S1).

**Table 2:** Predictive factors from Cox proportional analysis of postoperative overall survival (postoperative OS)

*N* = 271	HR (95% CI)	*P* value
Positive resection margin	1.8 (1.2–2.8)	< 0.01
TTS > 6 months^**a**^	1.6 (1.1–2.4)	< 0.01
Dominant liver lesion diameter	1.1 (1.0–1.1)	< 0.05
Age	1.02 (1.0–1.03)	< 0.05
Postoperative chemotherapy	0.63 (0.44–0.92)	< 0.05

^a^univariate analysis: HR 1.9 (1.4–2.7), *p* < 0.01

Additional analysis done to predict RFS also did not show association between TTS ≥ 6 months and RFS (HR 1.1, *p* = 0.48, Table S2). Exploratory analysis showed that patients with intermediate TTS were more likely to achieve no evidence of disease compared to short and long TTS (Table S3**)**. Finally, analysis related to the assessment of overall tumor burden among all three TTS intervals showed that patients with long TTS were more likely to have synchronous and bilateral liver metastases, more liver lesions, and more cycles of preoperative chemotherapy compared to intermediate TTS (all *p* < 0.01, Table S4). Patients with short TTS were more likely to have metachronous and unilateral liver metastases, fewer liver lesions, and fewer cycles of preoperative chemotherapy compared to intermediate TTS (all *p* < 0.01, Table S4).

## Discussion

In our study spanning a 16-year period of patients with resectable colorectal liver metastasis, the time to surgery (TTS) was associated with postoperative OS on multivariate analysis adjusting for disease characteristics but not definitively associated with overall survival from initial discovery of CRLM. However, intermediate TTS of 3 to 6 months had a trend toward better OS and improved 5-year OS (to 59%) and 8-year OS (to 43%) when compared to other TTS intervals. Long TTS of more than 6 months was specifically associated with worse postoperative OS, OS, and postoperative RFS, compared to other TTS intervals, but patients with long TTS also seemed to have higher disease burden and needed more preoperative chemotherapy prior to liver resection. All in all, these findings suggest that liver resection can be safely delayed for 3 months while delivering preoperative systemic therapy, but waiting beyond 6 months without a specific clinical reason may be associated with a decreased survival.

The ability to cure versus to simply delay relapse is difficult to differentiate using only the gold standard OS endpoint defined from initial discovery of CRLM to death. Patients with CRLM can have competing mortality risks (such as cardiovascular disease and other co-morbidities) over long-term follow-up, as observed in the final survival analysis of EORTC 40983 study [9]. Furthermore, multiple lines of effective systemic treatment options that prolong overall survival are now available at time of disease relapse [9]. For these reasons, measuring the hazard ratio longitudinally in a Kaplan-Meier analysis would not account for the differences in long-term survival that is more analogous to cures or known as the “tail-of-the-curve.” Specifically, our data shows an impressive 8-year OS of 43% among those with intermediate TTS compared to 29% in the short TTS and 18% in the long TTS.

Positive resection margin and size of liver lesion have already previously been identified as negative predictors of OS [11, 17, 18]. Our data identified similar negative predictors, which validates our dataset as consistent with known outcomes. The association of postoperative chemotherapy with improved OS, postoperative OS, and RFS indicates that chemotherapy remains imperative in the multidisciplinary treatment of patients with resectable CRLM, though the optimal timing and duration of chemotherapy have not yet been elucidated. Interestingly even in our univariate analysis, initial treatment response to first preoperative chemotherapy used was not associated with survival, which has been previously noted in the literature [19]. This is likely due to the fact that multiple active chemotherapy combinations with robust response rates of 54–65% could be used prior to or after surgery [20–23], but these regimens may only be suppressing rather than eliminating disease, at least for some patients. Therefore, post-treatment characteristics and predictive biomarkers of chemotherapy resistance may eventually be more useful prognostically. However, the time period from preoperative chemotherapy to resection could be useful to monitor treatment resistance and to unmask occult extrahepatic disease in order to better select appropriate patients with the correct tumor biology for which for liver resection is curative [24].

Less benefit was seen with long TTS was likely due to high tumor burden in this patient population and not because of prolonged chemotherapy causing harm, although there is clear evidence and concern for drug-induced liver toxicity from both oxaliplatin and irinotecan. Despite more liver lesions and larger liver metastasis that were associated with an increased number of cycles of preoperative chemotherapy in the long TTS group, more than 15% of patients were still able to achieve long-term survival. Similarly arguing that tumor biology is important, patients with short TTS were noted to have fewer and smaller liver lesions, but they still had early recurrences or disease-related deaths soon after definitive liver resection. In fact, their survival was not better than intermediate TTS. An ongoing trial in the Netherlands will specifically address whether preoperative chemotherapy is necessary for patients with clearly resectable CRLM but deemed high-risk by a validated recurrence risk score [25]. Our analysis gives an estimation of survival outcomes across short, intermediate, and long TTS that can be informative in assessing risk-benefit ratio in a given patient scenario. It is remains important to note that long-term survivors were observed across all TTS intervals, again emphasizing the importance of disease biology.

Conceptually, by deferring definitive liver resection for 3 months of perioperative chemotherapy, more imaging done over time could characterize the biologic behavior of colorectal cancer for that patient. We did not observe inferior survival in those with intermediate TTS, which was seen in an earlier cohort with solely immediately resectable CRLM and without any preoperative chemotherapy [15]. Serial imaging not only can measure treatment response but also characterize previously undetermined lesions and allows occult metastases to manifest. More sensitive imaging, such as PET/CT and MRI with eovist, could also be done in the interim, and in our dataset, about three quarters had preoperative PET scan, and a fifth had preoperative MRI of the liver prior to definitive liver resection. An ongoing international study, the Diffusion-Weighted MRI for Liver Metastasis (DREAM) study (NCT02781935), is assessing whether preoperative MRI is helpful in distinguishing whether CRLM is responding to preoperative chemotherapy in order to determine whether a specific liver lesion can be left behind (non-viable tumor) or needs to be resected at time of surgery.

### Limitations

This study has several limitations. First, the stratification of TTS by 3-month intervals may be suboptimal to detect clinically important differences in outcomes. This stratification scheme was ultimately chosen to be reflective of the EORTC 40983 study but nevertheless simplifies the varied duration in a retrospective analysis. TTS did also make the postoperative endpoints difficult to interpret due to lead time bias. Additionally, it may also have affected the interpretation of the overall survival due to immortal time bias. However, in the end, we feel all three survival outcome analyses together are complementary in confirming known validated factors as well as adding new data regarding TTS and postoperative chemotherapy. The number of cycles of chemotherapy was not used because there are a number of acceptable perioperative regimens that vary in cycle length (e.g., FOLFOX vs. CAPOX), and ultimately each treatment regimen is unique to patient scenario and preference. In addition, oxaliplatin- and irinotecan-based regimens were introduced in the late 1990s and early 2000s, thereby influencing the type of systemic therapy our patients were offered in the early time period of our cohort.

Second, TTS was directly associated with variables that quantify tumor burden (e.g., size and number of liver lesions), which also influenced survival and other treatment outcomes. While we were able to adjust these variables in our multivariate analyses, these confounding factors made detecting the potential association between TTS and OS difficult. Some of these factors, such as response rate and rate of positive margins, could not be definitively categorized from all charts reviewed. Regardless, we did see the same variables that were validated in other studies becoming statistically significant in our multivariate model.

Third, patients who did not undergo resection of CRLM were not captured in this study. It is possible that patients who were initially resectable may have become unresectable due to manifestation of occult metastatic disease. Starting with systemic therapy would seem desirable in this scenario in order to allow the disease biology to declare itself and spare patients futile surgery. Finally, data regarding recurrence and death were missing and censored early in some patients with shorter follow-up, resulting in median follow-up being shorter than the median OS and postoperative OS endpoints. Future randomized controlled trials could address all of these limitations.

## Conclusion

Given that TTS of 3 to 6 months had notable 5-year OS of 59% and 8-year OS of 43%, our findings suggest that there is likely an optimal intermediate time period from CRLM diagnosis to definitive liver resection. A period of perioperative chemotherapy is likely helpful to (1) establish the biologic behavior of CRLM, (2) permit a thorough serial radiologic assessment to assess manifestation of occult disease and chemotherapy resistance, (3) ensure multidisciplinary decision-making, and (4) downstage the extent of liver involvement for definitive resection with improved R0 resection. Understanding that some patients may have clinical situations that require beyond 6 months of preoperative chemotherapy, our study demonstrates that an optimal period associated with long-term survival likely exists between 3 to 6 months, for the majority of patients with CRLM. Our findings can potentially serve as the basis for future randomized-controlled trials testing novel neoadjuvant regimens for liver-limited metastatic colorectal cancer.

## Electronic supplementary material

ESM 1 (DOCX 21 kb)
