# Supplementary material for: Effect of Time to Surgery of Colorectal Liver Metastases on Survival
Source: J Gastrointest Cancer. 2020 Feb 22;52(1):169–76. doi: 10.1007/s12029-020-00372-5 (PMC7900034; doi:10.1007/s12029-020-00372-5)
Supplement: Supplementary file 1 — (DOCX 21 kb) [file 12029_2020_372_MOESM1_ESM.docx]

**Supplement**

**Table S1** Cox proportional analysis of overall survival (OS)...............…………………… **page 1**

**Table S2** Cox proportional analysis of recurrence-free survival (RFS)…………………… **page 2**

**Table S3** Comparison of time to surgery (TTS) between patients with no evidence of disease (NED) vs. with recurrence/death.……………………………………………..………….... **page 3**

**Table S4** Disease Characteristics of Short and Long TTS Patients Relative to Intermediate TTS ……………………………………………………………………………………………… **page 4**

| **Table S1.** Cox proportional analysis of overall survival (OS) | | |
| --- | --- | --- |
| **N=271** | **HR (95% CI)** | **P value** |
| Positive resection margin | 1.7 (1.1-2.6) | <0.01 |
| TTS > 6 months**^a^** | 1.1 (0.78-1.6) | 0.55 |
| Dominant liver lesion diameter | 1.1 (1.0-1.1) | <0.05 |
| Post-operative chemotherapy | 0.63 (0.43-0.91) | <0.05 |
| **^a^**univariate analysis: HR 1.4 (1.0-1.9), p=0.05 | | |

| **Table S2.** Cox proportional analysis of recurrence-free survival (RFS) | | |
| --- | --- | --- |
| **N=271** | **HR (95% CI)** | **P value** |
| Presence of lung metastasis | 2.1 (1.3-3.4) | <0.01 |
| Positive resection margin | 1.6 (1.1-2.2) | <0.01 |
| AJCC stage at initial diagnosis | 1.3 (1.1-1.6) | <0.01 |
| TTS > 6 months**^a^** | 1.1 (0.80-1.6) | 0.48 |
| Number of liver lesions | 1.1 (1.0-1.1) | <0.05 |
| Dominant liver lesion diameter | 1.1 (1.0-1.1) | <0.05 |
| Post-operative chemotherapy | 0.67 (0.48-0.92) | <0.05 |
| **^a^**univariate analysis: HR 1.7 (1.3-2.2), p<0.01 | | |

| **Table S3.** Comparison of time to resection (TTS) between patients with no evidence of disease (NED) vs. with recurrence/death | | | |
| --- | --- | --- | --- |
| **TTS (N=265)** | **NED** **(N=69)** | **Recurrence/ Death (N=196)** | **P value** |
| Short (<3 months) | 18 (26%) | 39 (20%) | <0.01 |
| Intermediate (3-6 months) | **32 (46%)** | 60 (31%) |  |
| Long (>6 months) | 19 (28%) | **97 (49%)** |  |

| **Table S4.** Disease Characteristics of Short and Long TTS Patients Relative to Intermediate TTS | | | | |
| --- | --- | --- | --- | --- |
| **N=281** | **Short TTS (N=64)** | **Intermediate TTS**  **(N=95)** | **Long TTS (N=122)** | **P value^b^** |
| **Lung metastasis** | 1 (1.6%) | 6 (6.3%) | 12 (9.8%) | 0.24 |
| **AJCC Stage at Diagnosis** |  | | | |
| I/II | 22 (35%) | 19 (20%) | 12 (9.8%) | <0.01 |
| III | 31 (50%) | 20 (21%) | 10 (8.2%) |  |
| IV | 9 (15%) | 56 (59%) | 100 (82%) |  |
| **Synchronous liver metastases** | 9 (14%) | 58 (61%) | 98 (80%) | <0.01 |
| **Bilateral liver metastases** | 11 (17%) | 37 (39%) | 70 (57%) | <0.01 |
| **Number of liver lesions^a^** (10 includes 10 or more) | 1 (1-10) | 2 (1-10) | 3 (1-10) | <0.01 |
| **Diameter of dominant liver lesion^a^** | 3.9 (1.0-12) | 3.2 (0.5-18) | 3.7 (0.2-21) | 0.07 |
| **Cycles of pre-operative chemotherapy^a^** | 0 (0-12**^c^**) | 6 (0-12) | 9 (0-72) | <0.01 |
| **^a^**reported as median (range)  **^b^**p-values indicate paired comparisons between all pairings of TTS groups; for non-significant p values the lowest is reported  **^c^**pre-operative chemotherapy cycles was counted for patients who developed liver metastasis during adjuvant FOLFOX chemotherapy | | | | |
